# Editorial: Cardiovascular Diseases Related to Diabetes and Obesity

**DOI:** 10.3389/fendo.2022.916142

**Published:** 2022-05-23

**Authors:** Mohanraj Rajesh, Ying Xin, Jamie L. Young, Lu Cai

**Affiliations:** ^1^Department of Pharmacology and Therapeutics, College of Medicine and Health Sciences, United Arab Emirates University, Al Ain, United Arab Emirates; ^2^Key Laboratory of Pathobiology, Ministry of Education, Jilin University, Changchun, China; ^3^Division of Gastroenterology, Hepatology & Nutrition, Department of Medicine, the University of Louisville School of Medicine, Louisville, KY, United States; ^4^Pharmacology and Toxicology, the University of Louisville School of Medicine, Louisville, KY, United States; ^5^Pediatric Research Institute, Department of Pediatrics, the University of Louisville School of Medicine, Louisville, KY, United States

**Keywords:** insulin resistance, diabetes, obesity, drug development, cardiomyopathy

Worldwide, the prevalence of obesity continues to rise unabated due to the rapid urbanization in the developed and developing countries and poor lifestyle habits. Obesity is also attributed to the development of major cardiovascular diseases (CVD), diabetes, arthritis, behavioral changes, depression, cancers and hepatic diseases. Therefore, it is of paramount significance that the development of obesity is thwarted by introducing behavioral changes (aimed at decreasing the consumption of high calorie food and inculcating the habit of regular physical activity). Ironically, these interventions do not benefit the majority of people who are morbidly obese. Furthermore, it is pertinent to note that emerging knowledge from clinical studies indicates that available pharmacological modes of therapy may not be sufficient to reduce the development of adverse CVD events in patients who are obese and have co-morbidities such as diabetes, hypertension or hepatic diseases such as non-alcoholic steatosis, non-alcoholic steatohepatitis or alcoholic steatohepatitis. Therefore, the objective of this special issue is to compile the recent clinical and pre-clinical studies which could expand our horizon in understanding the etiopathogenesis of these intertwined, multifactorial diseases.

Comprehensive reviews have highlighted the epidemiological forecasting of diabetes. Cheng et al., summarized that type 2 diabetes (T2DM) affected over 463 million people in 2019, and this number is expected to increase to 578 million by 2030 and 700 million by 2045; therefore, the priority objective is to identify all potential controllable risk factors for the development of diabetes with the purpose to have early screening and prevention. Based on results from the China Health and Retirement Longitudinal Study, increased serum uric acid is an important risk factor for the development of diabetes, in postmenopausal women. Venkatesan et al. tried to determine the prevalence of T2DM and estimate its heritability using family-based cohorts from three distinct Endogamous Ethnic Groups, representing Northern and Southern states of India. The heritability estimates of T2DM in these regions ranged from 30% to 82%. Other T2DM related traits (e.g., BMI, lipids, blood pressure) in these regions exhibited strong additive genetic influences, suggesting the high burden of T2DM in Indian Endogamous Ethnic Groups with significant and differential additive genetic influences on T2DM and related traits.

An elegant review by Y. Wu et al. highlights the critical role of mitochondria in the pathogenies of diabetes. Mitochondrial-derived peptides (MDPs) are recognized as short peptides formed by transcription and translation of the open reading frame site in human mitochondrial DNA. During stress the cell can use MDPs as a new type of reverse signal molecule to retrograde pass the signals to the nucleus where gene transcription synthesis is turned on to exert anti-inflammatory, anti-apoptotic signals and bolster the mitochondrial physiology, thereby mitigating the development of diabetes and CVD. Studies have shown a difference in serum apelin levels between individuals with and without diabetic and/or obesity, supporting the role of apelin in diabetes and obesity development and also implying the potential use of apelin as a clinical biomarker for diabetes and obesity. Therefore, additional clinical and experimental studies clearly supporting the physiological and pathophysiological roles of the apelin–APJ system in glucose and lipid metabolism, particularly with its signaling pathways, is warranted (Li et al.).

In terms of diabetic complications, Chen et al. analyzed patients with acute myocardial infarction (AMI), admitted into a coronary care unit with follow-up of ≥1 year based on two cohorts of MIMIC-III (Medical Information Mart for Intensive Care III) and CIN (Cardiorenal Improvement Registry) in the United States and China, and found that AMI patients with diabetes have a significantly higher 30-day mortality and increased 1-year mortality than AMI patients without diabetes. Similarly, Du et al., reported that insulin resistance is associated with increased risk of CVD development in adolescent subjects with diabetes. L. Wu et al. described that Apolipoprotein E polymorphisms are associated with the development of CVD in patients with or without T2DM. By utilizing bioinformatic analysis tools and validation with db/db T2DM mouse heart tissue, Huang et al. revealed that calpain small subunit 1(CAPNS1) is highly expressed in the heart of T2DM db/db mice and this was significantly decreased in the heart of T2DM patients with SARS-CoV-2 infection, suggesting a novel target in mitigating the adverse effects of SARS-Cov-2 infection. Although this is a novel and interesting hypothesis developed based on bioinformatic approach, the precise clinical significance is blurred and should be confirmed by additional preclinical and clinical studies.

Aberrant endothelial function in patients with T2DM is closely associated with the development of CVD. Flow-mediated dilation (FMD) is a noninvasive tool for evaluating endothelial function, which typically examines changes in the brachial artery diameter in response to ischemia using ultrasound Doppler. By evaluating FMD in patients with T2DM, Wang et al. performed a network meta-analysis to explore the improvement of endothelial function with antidiabetic drugs and found that glucagon-like peptide-1 receptor (GLP-1R) agonists, sodium glucose co-transporter-2 inhibitor and thiazolidinedione exhibited favorable effects in improving the endothelial function in T2DM patients. Consistently, Dardano et al. also reported the efficiency and safety of GLP-1R agonist in mitigating adverse effects of CVD in a T2DM patient living with HIV. In addition, the pre-clinical study indicated the protective effects of GLP-1R agonists treatment in mitigating the development of diabetic cardiomyopathy (El-Shafey et al.). Last, the meta-analysis by Sun et al. revealed that sesamin dietary supplementation improved blood pressure and serum lipid profiles, and postulated sesamin as a supplement therapy or health supplement to prevent the development of CVDs.

In sum, the aforementioned articles published in this Research Topic highlight the multifaceted etiopathogenic aspects of closely connected diseases such as obesity and diabetes. It also sets the stage for delving further in deciphering the crucial link between obesity and diabetes. This could also help in unraveling the selective and specific biomarkers and/or pharmacological targets, aiding in the management of these debilitating chronic diseases.

## Author Contributions

MR and LC wrote the first draft. MR, JY, YX, and LC revised and approved the final submitted version.

## Conflict of Interest

The authors declare that the research was conducted in the absence of any commercial or financial relationships that could be construed as a potential conflict of interest.

## Publisher’s Note

All claims expressed in this article are solely those of the authors and do not necessarily represent those of their affiliated organizations, or those of the publisher, the editors and the reviewers. Any product that may be evaluated in this article, or claim that may be made by its manufacturer, is not guaranteed or endorsed by the publisher.

